# Islands in an Obesogenic Ocean: A Multiscale Spatial Analysis of School Neighborhood Food Environments in Michigan

**DOI:** 10.3390/ijerph23070835

**Published:** 2026-06-25

**Authors:** Gang Xu

**Affiliations:** Department of Geography and Sustainable Planning, Grand Valley State University, Allendale, MI 49401, USA; xug@gvsu.edu

**Keywords:** school neighborhood, food environment, mRFEI, multiscale spatial analysis, urban–rural disparities, accessibility, availability, unhealthful food outlets, Michigan

## Abstract

**Highlights:**

**Public health relevance—How does this work relate to a public health issue?**

- School neighborhood food environments in Michigan are characterized by widespread exposure to unhealthful food outlets, a key driver of obesogenic environments affecting children’s dietary behaviors.
- This study addresses a critical evidence gap by providing the first statewide multiscale assessment of school neighborhood food environments in Michigan.

**Public health significance—Why is this work of significance to public health?**

- This study reveals strong spatial clustering and uneven distribution of fast food restaurants and convenience stores near schools, with significantly higher exposure in urban than rural areas.
- This study demonstrates the value of integrating healthfulness (mRFEI), availability, and accessibility across multiple spatial scales to better capture environmental risk.

**Public health implications—What are the key implications or messages for practitioners, policy makers and/or researchers in public health?**

- Findings support place-based policy interventions (e.g., zoning, outlet regulation, healthy food incentives) targeting high-exposure school neighborhoods.
- This study highlights the importance of multiscale and multidimensional approaches for research, planning, and policy development aimed at improving school neighborhood food environments.

**Abstract:**

This study examines the retail food environment surrounding public schools in Michigan using a multiscale, multidimensional framework. A cross-sectional spatial analysis integrates relative healthfulness (modified Retail Food Environment Index, mRFEI), availability (outlet counts), and accessibility (network-based walking time) across school districts, census tracts, block groups, and school-centered buffers. The analysis includes 3530 public schools, 7680 fast food restaurants, and 2065 convenience stores. Results show pronounced spatial heterogeneity and clustering of unhealthful outlets (Nearest Neighbor Index = 0.284, *p* < 0.001), with many located near schools. Approximately 34% of schools are within a 10 min walk of a fast food restaurant, increasing to 65% within a 20 min walk. Urban schools face significantly greater exposure—2.27–2.80 times more fast food outlets and shorter walking times than rural schools (*p* ≤ 0.002)—with consistent gradients across city, suburban, town, and rural contexts. Overall, school neighborhood food environments are highly structured, obesogenic, and inequitable. By integrating multiple spatial scales and complementary measures of food environments, this study advances food environment research and provides policy-relevant evidence for targeted, place-based interventions to improve access to healthier food around schools.

## 1. Introduction

Poor dietary quality among children and adolescents remains a major public health concern in the United States. A substantial proportion of youth fail to meet national dietary recommendations, with inadequate consumption of fruits, vegetables, and whole grains and frequent intake of energy-dense, nutrient-poor foods [1,2,3,4]. These dietary patterns are strongly associated with adverse health outcomes, including childhood overweight and obesity, which have increased markedly over recent decades [5,6]. Despite federal efforts to improve school meal quality through programs such as the National School Lunch Program and School Breakfast Program, children’s overall nutritional status remains suboptimal [7].

Children’s dietary behaviors are shaped by a complex interplay of influences operating across multiple levels, consistent with socio-ecological models of health behavior [8,9,10]. Among these influences, the neighborhood food environment—defined by the availability, accessibility, and relative healthfulness of food outlets—plays a critical role in shaping dietary behaviors and health outcomes [11,12,13]. The school neighborhood food environment is particularly important, as schools and their surrounding areas constitute primary daily activity spaces where children are repeatedly exposed to food options [14,15]. In Michigan alone, more than 1.38 million children were enrolled in public schools during the 2024–2025 academic year [16], highlighting the scale at which school-centered environments may influence population-level dietary behaviors. Frequent exposure to nearby food outlets, coupled with increasing autonomy in food purchasing—particularly among adolescents—may significantly influence food preferences, consumption patterns, and diet-related health outcomes [14,15,17,18].

A growing body of research demonstrates that the spatial characteristics of school neighborhood food environments are related to children’s eating habits and health outcomes [12,19,20]. Evidence consistently shows that greater availability and proximity of unhealthful food options—particularly fast food restaurants and convenience stores—near schools are associated with increased consumption of high-calorie foods and lower intake of fruits and vegetables [15,17,21,22,23]. Additionally, higher exposure to unhealthful outlets has been linked to increased body mass index and a greater prevalence of overweight and obesity among children and adolescents [14,18,24,25]. Importantly, emerging evidence suggests that the external food environment may moderate the effectiveness of school-based nutrition policies. Even when schools implement interventions to promote healthy eating, the presence of nearby unhealthful food outlets may undermine these efforts by providing convenient and appealing alternatives, thereby influencing student food choices beyond the school setting [26].

Although school meal programs play an important role in shaping students’ dietary intake, food environments surrounding schools remain relevant because adolescents are exposed to nearby food outlets before and after school, during commuting, and through other school-related activities. School neighborhood food environments therefore represent important contexts of potential exposure that may influence food purchasing opportunities, dietary behaviors, and overall nutritional environments. Recent evidence suggests that exposure to food outlets encountered throughout daily activity spaces can shape food-related behaviors across multiple contexts of adolescents’ daily lives [15,19,23].

Methodologically, prior studies have employed a range of approaches to define and measure school neighborhood food environments. Two dominant approaches include the use of administrative units (e.g., census tracts or census block groups) and spatial buffers around schools [11,12,27]. Administrative units capture broader contextual characteristics of the areas in which schools are embedded and are frequently used for population-level assessment, planning, and policy development. In contrast, school-centered network buffers provide a more behaviorally relevant representation of potential exposure by accounting for pedestrian travel pathways and the immediate environments surrounding schools [28,29]. Because food environments may differ across geographic contexts and levels of aggregation, examining multiple spatial scales can provide a more comprehensive understanding of exposure than reliance on a single geographic scale, which may capture only a subset of the environmental contexts surrounding schools.

In addition to spatial scale, food environments can be characterized using multiple measures of exposure. Common measures include availability (e.g., counts and density of outlets) and accessibility (e.g., distance or travel time to the nearest outlet), with density and proximity being the most widely used indicators [11,12,15,30,31,32]. Composite indices such as the modified Retail Food Environment Index (mRFEI) have been increasingly used to quantify the relative balance of healthy and unhealthful food outlets within a given area [33,34,35,36].

Despite substantial progress, several important gaps remain in the literature. First, many studies assess school food environments using a single geographic scale, limiting understanding of how food environment characteristics vary across broader contextual environments and immediate school-centered activity spaces. Second, although individual measures such as outlet density, proximity, and food environment indices are widely used, fewer studies evaluate these complementary measures within a unified analytical framework. Third, although urban–rural disparities in food environments have been documented, comprehensive analyses examining gradients across multiple urbanicity categories remain limited. Finally, no study to date has conducted a statewide multiscale assessment of school neighborhood food environments across Michigan [37,38,39,40].

Addressing these gaps is important for both research and practice. Understanding how food environment characteristics vary across spatial scales, exposure measures, and urbanicity contexts can improve the identification of inequities and inform more targeted public health interventions. Such evidence can support land-use planning, zoning policies, healthy food retail initiatives, and other place-based strategies aimed at creating healthier school neighborhood environments.

Therefore, the aim of this study is to conduct a multiscale spatial analysis of the retail food environment around public schools in Michigan. Specifically, this study: (1) evaluates the relative healthfulness of food environments using mRFEI across school districts, census tracts, and block groups; (2) assesses the availability and accessibility of unhealthful food outlets—defined as fast food restaurants and convenience stores—using multiple network-based walking-time buffers (10, 15, and 20 min thresholds); and (3) examines variation across urbanicity categories, including city, suburban, town, and rural settings. By integrating multiple spatial scales, complementary measures of food environment exposure, and detailed urbanicity classifications within a statewide analysis, this study provides a comprehensive assessment of school neighborhood food environments in Michigan and offers evidence to inform public health policy and planning.

The remainder of this paper proceeds as follows: Section 2 describes the study design and methods. Section 3 presents the findings. Section 4 discusses their implications for research and practice. Section 5 concludes.

## 2. Materials and Methods

### 2.1. Study Design and Study Area

This study is a cross-sectional spatial analysis examining the retail food environment surrounding public schools in Michigan, USA. The study area includes all public schools and their surrounding neighborhoods across diverse geographic contexts ranging from highly urbanized to remote rural settings. The analysis included 3530 public schools across 541 school districts, encompassing 1792 census tracts (hereafter “school tracts”) and 2303 census block groups (“school block groups”) where schools are located. A total of 7680 fast food restaurants and 2065 convenience stores were included in the analysis.

A multiscale spatial analytical framework was employed to capture variation in food environments across multiple geographic levels. Analyses were conducted at four spatial scales: (1) school districts, (2) school tracts, (3) school block groups, and (4) school-centered network-based walking-time buffers. The inclusion of multiple geographic scales allowed assessment of food environments across broader contextual environments and immediate school-centered environments, facilitating comparison of patterns observed at different levels of spatial aggregation.

The analytical workflow consisted of two complementary components: (1) area-based analysis of the relative healthfulness of food environments using the modified Retail Food Environment Index (mRFEI) and (2) school-level analysis of exposure and accessibility to unhealthful food outlets. This integrated approach aligns with recent food environment research emphasizing multiscale and multidimensional analysis [15].

### 2.2. Data Sources and Food Outlet Classification

Public school locations and school district boundaries were obtained from the National Center for Education Statistics (NCES) Education Demographic and Geographic Estimates (EDGE) program [41]. Food outlet data were derived from Esri Business Analyst—U.S. 2025 Dataset [42], supplemented with farmers markets, on-farm markets, and food hubs from the USDA Local Food Directory [43].

Food outlets were classified using North American Industry Classification System (NAICS) codes into healthy and unhealthful categories. Healthy food retailers included supermarkets, grocery stores, warehouse clubs, specialty food retailers, and USDA-listed local food sources, while unhealthful outlets included fast food restaurants and convenience stores. This classification approach is widely used in food environment research [33,44].

### 2.3. Multiscale Analysis of Food Environment Healthfulness

The modified Retail Food Environment Index (mRFEI) was used to quantify the relative availability of healthy versus unhealthful food outlets [33]. The mRFEI represents the proportion of healthy food retailers relative to the total number of food retailers within a defined area and is expressed as a percentage:

(1) $$mRFEI=\left(\frac{Healthy\;Food\;Retailers}{Total\;Food\;Retailers}\right)\times 100$$

Values range from 0 (no healthy food retailers) to 100 (all food retailers are classified as healthy), with higher values indicating a healthier retail food environment. mRFEI scores were calculated at three spatial scales: school districts, school tracts, and school block groups.

Spatial clustering of school district mRFEI scores was examined using the Getis–Ord Gi* statistic. Hot spot and cold spot analyses were conducted using the Optimized Hot Spot Analysis tool in ArcGIS Pro, version 3.2 [45]. This tool identifies statistically significant spatial clusters while automatically determining an optimal analysis scale based on Incremental Spatial Autocorrelation. The optimized workflow reduces subjectivity associated with selecting a fixed distance threshold and provides a data-driven approach for identifying the spatial scale at which clustering is most pronounced. To account for multiple testing and spatial dependence, the False Discovery Rate (FDR) correction procedure was applied. Districts with significantly high mRFEI values were identified as hot spots, whereas districts with significantly low mRFEI values were identified as cold spots. The resulting Gi* z-scores and associated *p*-values were used to map statistically significant spatial clusters of food environment healthfulness across Michigan [46].

To assess within-district variability in food environment healthfulness, the coefficient of variation (CV) was calculated using block group–level mRFEI scores aggregated to the district level:

(2) $$CV=\left(\frac{\sigma }{\mu }\right)\times 100$$

where *σ* represents the standard deviation, and *μ* represents the mean mRFEI. Higher CV values indicate greater spatial heterogeneity in food environment healthfulness among block groups within a school district, reflecting potential intra-district inequalities.

All mRFEI-related outputs were mapped to visualize spatial patterns across Michigan. To facilitate comparison across geographic scales, identical mRFEI class intervals were applied to all thematic maps of food environment healthfulness.

### 2.4. School-Level Spatial Analysis

Spatial relationships between public schools and unhealthful food outlets were examined through a series of complementary analyses. School locations were first overlaid with fast food restaurants and convenience stores to visualize co-location patterns. The spatial distribution of unhealthful food outlets was further evaluated using Nearest Neighbor Analysis (NNA) to determine whether outlets were clustered, randomly distributed, or dispersed [47].

To assess school exposure to unhealthful food outlets, network-based walking-time service areas of 10 and 20 min were generated around each unhealthful outlet (hereafter “walkable service areas”). Schools located within walkable service areas were identified through spatial joins. Network-based buffers were used because they more accurately represent realistic accessibility and pedestrian travel behavior than simple Euclidean buffers [27]. Walking-time thresholds were based on commonly used pedestrian-accessibility measures in food environment and school neighborhood research [11,48]. These thresholds were not intended to represent Michigan-specific behavioral cutoffs but rather to capture progressively larger school neighborhood contexts and facilitate comparison with prior studies.

Availability of unhealthful food outlets near schools was assessed using network-based buffers of 10, 15, and 20 min constructed around each school (hereafter “walkable school zones”). Counts of fast food restaurants and convenience stores within each walkable school zone were calculated. The use of multiple buffer thresholds enables a multiscale assessment of food outlet availability across increasingly expansive school neighborhood environments and reduces potential bias associated with reliance on a single neighborhood boundary definition [48].

Accessibility was further assessed by calculating the network-based walking time from each school to the nearest unhealthful food outlet, capturing the minimum effort required to access these food options. This proximity-based measure complements availability-based metrics and is widely used in food environment research [12].

Schools were stratified using the NCES school locale classification system into urban–rural and four-category (city, suburban, town, and rural) classifications, a framework commonly used in school neighborhood environment research [29].

### 2.5. Statistical Analysis

Differences in accessibility were assessed using Welch’s *t*-tests and Welch’s ANOVA, followed by Games–Howell post hoc tests for pairwise comparisons. These procedures were selected because they are robust to unequal variances and unequal group sizes. Statistical significance was set at *p* < 0.05, and effect sizes were reported using Cohen’s *d* and partial eta squared (*ηp*^2^). Median and interquartile range (Q1–Q3) statistics were also calculated to facilitate interpretation of skewed walking-time distributions. Levene’s tests were conducted to assess homogeneity of variances, and Mann–Whitney *U* tests were performed as nonparametric sensitivity analyses.

Differences in outlet availability were analyzed using negative binomial regression models to account for overdispersion in count data. The models were used to describe differences in outlet availability across urbanicity categories rather than estimate the independent effect of urbanicity after adjustment for demographic, socioeconomic, or other contextual covariates. The dependent variables were the counts of fast food restaurants and convenience stores within walkable school zones. No offset term was included; therefore, incidence rate ratios (IRRs) represent relative differences in outlet counts rather than outlet densities. Results are reported as IRRs with 95% confidence intervals.

All spatial analyses were conducted using ArcGIS Pro, version 3.2 [45], and statistical analyses were performed using IBM SPSS Statistics, version 30.0 [49].

## 3. Results

### 3.1. Multiscale Patterns of Food Environment Healthfulness (mRFEI)

**Figure 1:**
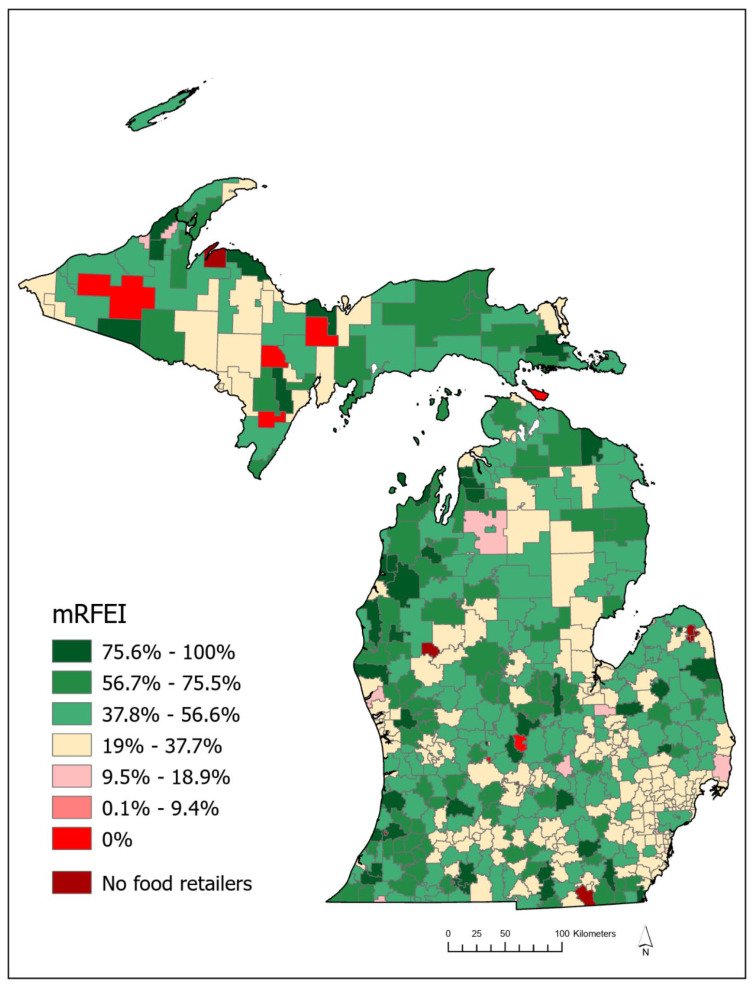
The modified Retail Food Environment Index (mRFEI) scores by school districts in Michigan.

**Figure 2:**
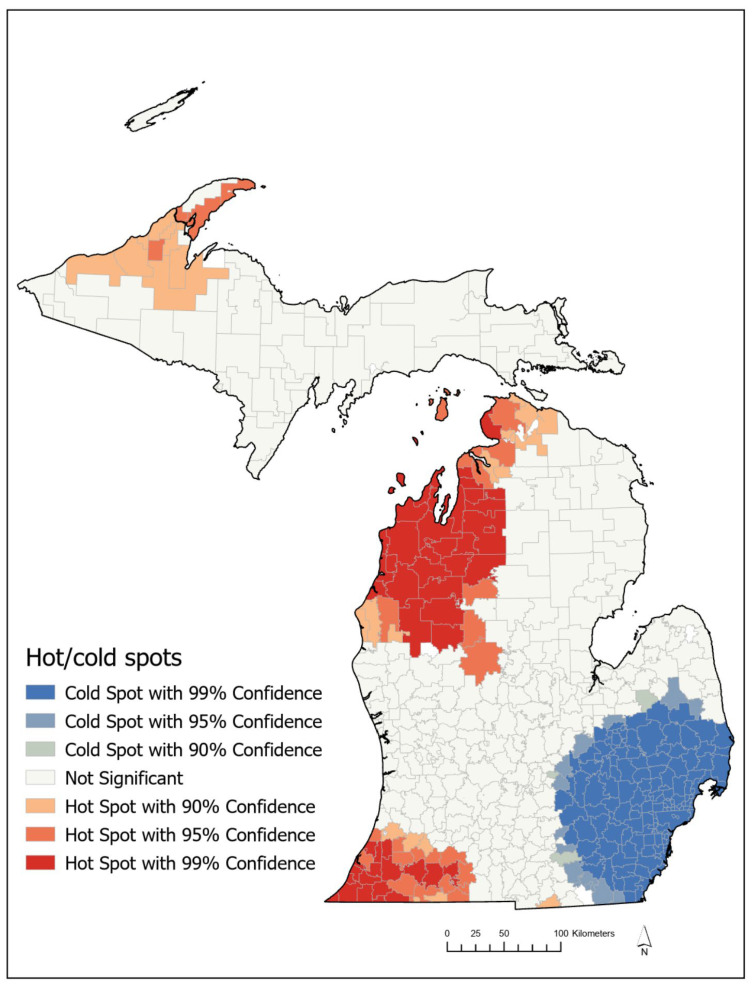
Hot spots and cold spots of mRFEI scores at the school district level (Getis-Ord Gi*) in Michigan.

**Figure 3:**
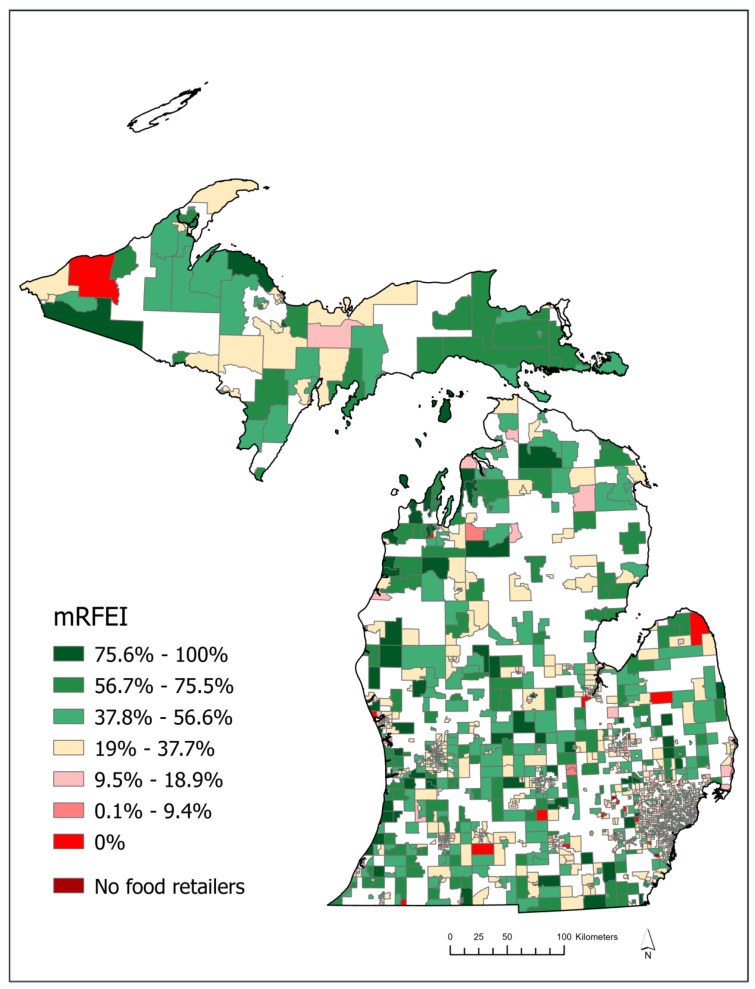
The modified Retail Food Environment Index (mRFEI) scores by census tracts where Michigan public schools are located.

**Figure 4:**
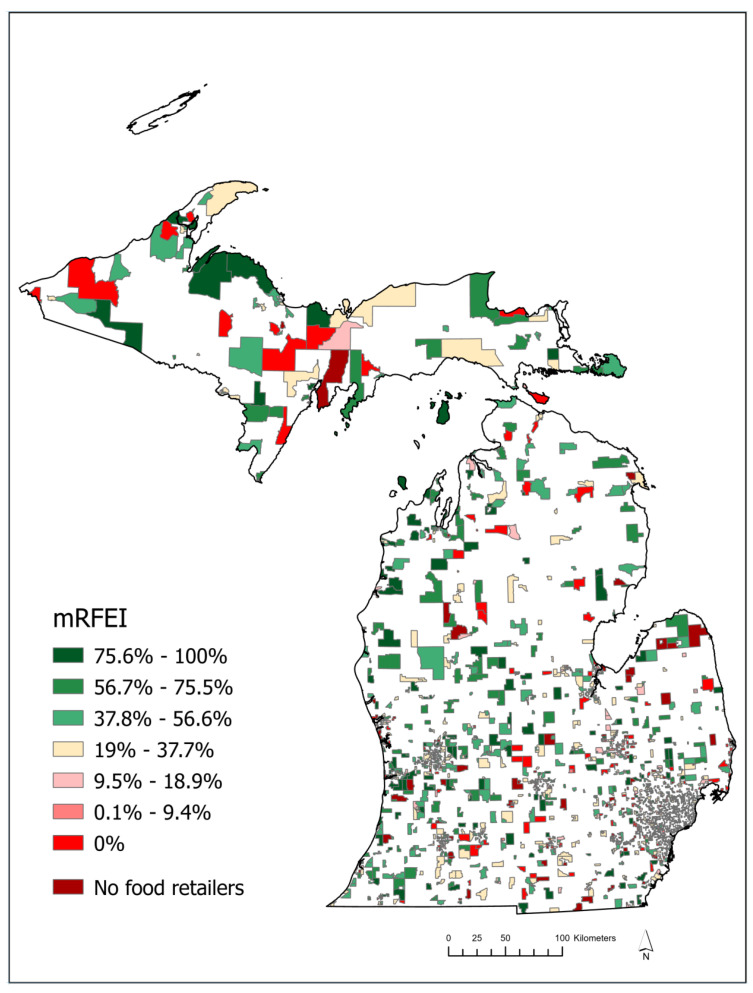
The modified Retail Food Environment Index (mRFEI) scores by census block groups where Michigan public schools are located.

**Figure A1:**
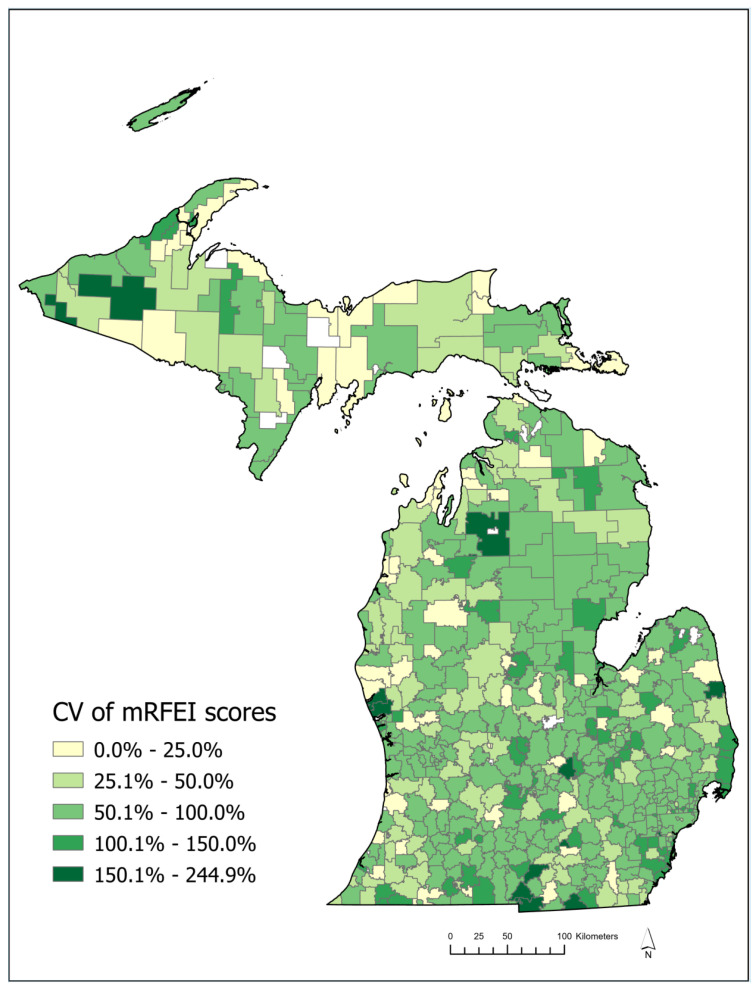
The coefficient of variation (CV) of the modified Retail Food Environment Index (mRFEI) scores by school districts in Michigan.

School food environment healthfulness was examined across district, census tract, and census block group scales. Figure 1 presents district-level mRFEI patterns, Figure 2 identifies statistically significant district-level hot spots and cold spots of mRFEI, and Figure 3 and Figure 4 present progressively finer-scale mRFEI patterns at the census tract and census block group levels, respectively. District-level variation in mRFEI is further summarized using the coefficient of variation (CV) in Figure A1.

The spatial distribution of modified Retail Food Environment Index (mRFEI) scores across Michigan demonstrates substantial geographic variability in the healthfulness of school neighborhood food environments. At the school district level (Figure 1), mRFEI values ranged from 0 (i.e., no healthy food retailers) to 100 (i.e., all food retailers are classified as healthy), with most districts falling within moderate ranges, indicating a mixture of healthy and unhealthful food outlets. Higher mRFEI values were more prevalent in western Michigan, the northern Lower Peninsula, and parts of the Upper Peninsula, whereas lower values were concentrated in the central and southeastern Lower Peninsula, particularly in metropolitan regions.

A small number of districts contained no food retailers, representing cases of extremely limited food availability. Overall, the distribution of mRFEI values indicates considerable spatial heterogeneity in food environment healthfulness across the state.

Using the optimized Getis–Ord Gi* workflow, statistically significant hot spots and cold spots of mRFEI at the school-district level were identified across Michigan (Figure 2). Cold spots (low mRFEI values) were more numerous and spatially concentrated than hot spots, with the most prominent cluster located in Southeast Michigan. In contrast, hot spots were fewer and more dispersed, with the largest cluster observed in Northwest Michigan.

Substantial intra-district variability was observed in mRFEI values (Figure A1). The coefficient of variation (CV) ranged from 0% to 245%, with most districts exhibiting moderate variability (50–100%). Lower variability was more common in northern and rural regions, whereas higher variability was concentrated in central and southeastern Michigan.

At finer spatial scales, the food environment exhibited increased fragmentation. At the census tract level (Figure 3), moderate mRFEI values predominated, with clusters of low values in urban cores—particularly in Southeast Michigan—and remote rural areas. Some school tracts contained no food retailers altogether. This pattern became more pronounced at the block group level (Figure 4), where low and zero mRFEI values were more spatially concentrated in urbanized regions, while areas without food retailers were more prevalent in rural parts of northern and central Michigan and the Upper Peninsula.

### 3.2. Spatial Clustering of Unhealthful Food Outlets

**Figure 5:**
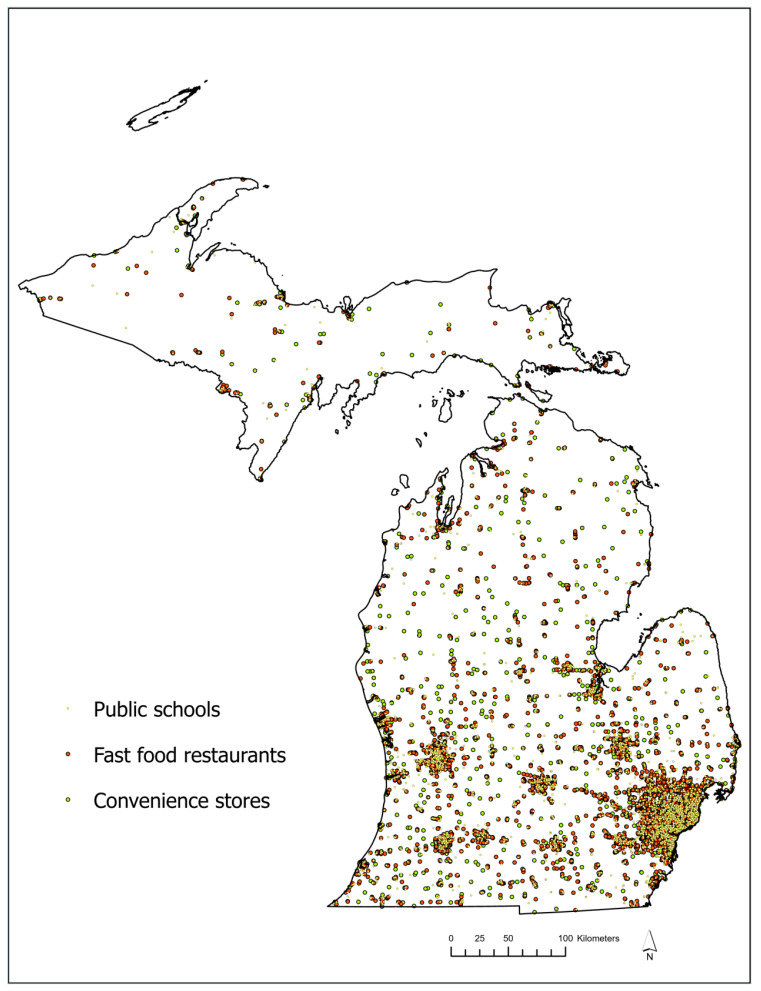
Co-location patterns of public schools and unhealthful food outlets in Michigan.

Fast food restaurants and convenience stores exhibited strong spatial clustering across Michigan, with the highest densities located in the southern Lower Peninsula, particularly in urban areas and along major transportation corridors. In contrast, northern and rural regions showed substantially lower outlet densities (Figure 5).

Spatial overlay analysis indicated a clear correspondence between public school locations and clusters of unhealthful food outlets. Many schools in urbanized regions were located within or adjacent to dense concentrations of fast food restaurants and convenience stores. This co-location pattern was more pronounced for fast food restaurants, which were more numerous and spatially extensive than convenience stores.

Nearest Neighbor Analysis confirmed that unhealthful food outlets were significantly clustered (Table A1). The Nearest Neighbor Index (NNI) indicated strong clustering for all unhealthful outlets combined (NNI = 0.284, *p* < 0.001), with fast food restaurants exhibiting the most pronounced clustering (NNI = 0.248) and convenience stores showing moderate clustering (NNI = 0.575).

### 3.3. Exposure Within Walkable School Environments

Network-based analyses demonstrated substantial exposure of Michigan public schools to unhealthful food outlets within walkable distances (Table A2). Approximately 34% of schools were located within a 10 min walk of a fast food restaurant, increasing to approximately 65% within a 20 min walk. For convenience stores, approximately 22% of schools were within a 10 min walk, increasing to more than 51% within a 20 min walk.

The distribution of unhealthful food outlets within walkable school zones showed a clear distance-dependent pattern (Table A3). Approximately one-third of unhealthful food outlets were located within 10 min walking distances of schools, more than half within 15 min, and over two-thirds within 20 min. Across all buffer distances, fast food restaurants consistently outnumbered convenience stores.

### 3.4. Urbanicity-Based Disparities in Availability and Accessibility

Consistent urban–rural gradients were observed in both availability and accessibility of unhealthful food outlets.

Availability analyses (Table 1 and Table 2) indicated that urban schools were surrounded by substantially greater numbers of both fast food restaurants and convenience stores within school-centered walkable zones than rural schools. Negative binomial regression models showed that urban schools had between 2.27 and 2.80 times as many fast food outlets and 1.25 to 1.87 times as many convenience stores within 10–20 min walking buffers (*p* ≤ 0.002; 95% confidence intervals entirely above 1). These differences increased with buffer distance.

Across four urbanicity categories, outlet availability followed consistent gradients. At 10 and 15 min buffer distances, the pattern was City > Town > Suburban > Rural, while at the 20 min distance, it shifted slightly to City > Suburban > Town > Rural. These differences were statistically significant across all models (*p* < 0.001). Across all categories and distances, fast food outlets were more numerous than convenience stores.

Accessibility analyses (Table 3 and Table 4) showed that urban schools had significantly shorter walking times to the nearest unhealthful food outlet than rural schools. Mean walking times to the nearest fast food restaurant were 15.0 min for urban schools and 38.5 min for rural schools (*t* = −16.64, *p* < 0.001, *d* = 0.68). For convenience stores, mean walking times were 21.2 min for urban schools and 48.9 min for rural schools (*t* = −16.29, *p* < 0.001, *d* = 0.66).

Across four urbanicity categories, differences in walking time were statistically significant for both fast food (*F* = 164.11, *p* < 0.001, *ηp*^2^ = 0.171) and convenience stores (*F* = 166.24, *p* < 0.001, *ηp*^2^ = 0.128). Post hoc comparisons indicated systematic gradients in walking time, with City < Town < Suburban < Rural for fast food and City < Suburban < Town < Rural for convenience stores (all *p* < 0.05). Detailed Games–Howell pairwise comparisons are provided in Table A4.

Rural areas exhibited greater variability in walking times, with standard deviations reaching approximately 60–70 min, indicating more heterogeneous access compared with urban areas.

Median (Q1–Q3) statistics for walking times are provided in Table A5 and demonstrate patterns consistent with the mean-based analyses reported in Table 3 and Table 4. Robustness checks, including Levene’s tests and Mann–Whitney *U* tests, are reported in Table A6 and supported the robustness of the primary findings.

### 3.5. Summary of Key Findings

Across all analyses, the retail food environment surrounding public schools in Michigan exhibited pronounced spatial variation. Unhealthful food outlets were strongly clustered and frequently located near schools, particularly in urban areas. Network-based analyses indicated substantial exposure to unhealthful food outlets within walkable distances, with exposure increasing as buffer distance expanded.

Clear and consistent urban–rural gradients were observed across all measures. Urban schools were characterized by both greater availability of and closer proximity to unhealthful food outlets, whereas rural schools had lower availability but longer and more variable travel times.

## 4. Discussion

This study provides a holistic, nuanced assessment of the nutritional landscape surrounding public schools in Michigan. Across all analyses, a consistent pattern emerges: school neighborhood food environments are highly uneven, spatially structured, and characterized by substantial exposure to unhealthful food outlets. Evidence from multiple measures—relative healthfulness (mRFEI), outlet availability, proximity, and spatial clustering—converges to indicate that many Michigan public schools are situated within environments where unhealthful food options are both abundant and readily accessible.

By integrating multiple spatial scales and complementary exposure measures, this study extends prior research and demonstrates that food environments surrounding Michigan schools are not only heterogeneous but systematically structured in ways that shape the intensity and immediacy of exposure.

### 4.1. Multiscale Inequities in Food Environment Healthfulness

A central contribution of this study is the demonstration of pronounced spatial inequities in food environment healthfulness across multiple geographic scales. The clustering of low mRFEI values in southeastern Michigan, particularly in metropolitan regions, indicates persistent regional disparities in the relative balance of healthy and unhealthful food outlets. In contrast, higher mRFEI values observed in western and northern regions are more spatially dispersed and less regionally dominant.

At finer spatial scales, substantial intra-district variability further highlights localized inequities. The wide range of coefficient of variation (CV) values indicates that school districts often contain neighborhoods with markedly different food environments. These findings underscore the value of multiscale analysis because different geographic scales reveal distinct aspects of school food environments. District-level patterns highlighted broad regional disparities, whereas finer-scale analyses revealed substantial variation within districts that would not be apparent from larger administrative units alone.

Together, these results demonstrate that food environment inequities operate simultaneously across broader contextual environments and localized school-centered settings. These findings are consistent with prior research documenting spatial inequalities in food environments across geographic contexts [50].

### 4.2. Convergence of Exposure: Availability, Accessibility, and Clustering

The findings reveal a strong convergence across multiple measures of food environment exposure. Availability analyses indicate that unhealthful food outlets are highly concentrated around schools, particularly in urban areas, with fast food restaurants consistently more prevalent than convenience stores. Accessibility analyses show that a substantial proportion of Michigan schools are located within short walking distances of unhealthful outlets, indicating that exposure is not only widespread but behaviorally relevant. In addition, spatial clustering analyses confirm that these outlets are not randomly distributed but are concentrated in specific areas.

This convergence suggests that many schools are embedded within environments where unhealthful food outlets are numerous, nearby, and spatially clustered. Such configurations intensify environmental exposure and increase the likelihood of repeated interaction with these food sources during daily school-related activities, including travel to and from school and other routine activities occurring beyond school meal periods. This pattern aligns with prior studies demonstrating that combined measures of availability and accessibility provide a more comprehensive understanding of food environment exposure than single measures alone [12].

### 4.3. Urban–Rural Dual Burden of Food Environments

A key finding of this study is the identification of a dual burden of food environment inequity operating through distinct mechanisms in urban and rural contexts. In urban areas, high outlet availability and strong clustering create conditions characterized by concentrated exposure to unhealthful food options. The results indicate that urban schools have not only significantly higher numbers of nearby outlets but also shorter walking times to these outlets, reflecting both the intensity and immediacy of exposure.

In contrast, rural areas exhibit a different form of spatial disadvantage. Lower outlet availability and, in some cases, the absence of nearby food retailers reflect limited opportunities for access. At the same time, greater variability in walking times indicates more heterogeneous and less predictable accessibility. This combination of scarcity and spatial dispersion suggests that food environments surrounding rural schools are characterized by constraints on access rather than excess exposure.

These findings indicate that inequities in school neighborhood food environments are not uniform but instead reflect distinct structural conditions across the urban–rural continuum. Addressing these disparities requires differentiated approaches that account for the underlying mechanisms shaping food access in each context. These results are consistent with existing literature documenting urban–rural disparities in food access and availability [34].

### 4.4. Urbanicity Gradients and Scale-Dependent Exposure

The study further demonstrates that food environment exposure follows clear and systematic gradients across urbanicity categories. Consistent patterns observed across both availability and accessibility measures indicate that city environments represent the highest levels of exposure, followed by suburban and town contexts, with rural areas exhibiting the lowest overall exposure.

Importantly, the magnitude of these differences increases with spatial scale. As buffer distances expand from 10 to 20 min, disparities in outlet availability become more pronounced, indicating that exposure differences accumulate across broader activity spaces. This scale-dependent pattern underscores the importance of using multiple spatial thresholds when assessing school neighborhood food environments, as single-distance measures may underestimate the extent of exposure [29].

The findings also reveal spatial nuance within intermediate urbanicity categories. The relative positioning of suburban and town contexts varies across distances, indicating that these environments do not follow a uniform pattern. In contrast, rural areas consistently exhibit lower availability but greater variability in accessibility, reinforcing their distinct spatial characteristics.

Furthermore, the differing positions of suburban and town schools across availability and accessibility measures suggest distinct spatial configurations of food outlets. Although suburban schools generally exhibited greater outlet availability at larger buffer distances, town schools often demonstrated comparable or shorter walking times to the nearest outlet. This pattern suggests that town environments may contain fewer outlets overall yet have outlets located relatively close to schools, whereas suburban environments may contain larger numbers of outlets distributed across broader commercial corridors and activity spaces. These findings highlight how availability and accessibility capture complementary aspects of food environments and reinforce the importance of using multiple measures when assessing exposure to unhealthful food outlets.

### 4.5. Policy and Public Health Implications

The findings of this study have several important implications for public health policy and intervention. First, the multiscale nature of exposure indicates that strategies limited to immediate school surroundings may be insufficient. Interventions should consider broader neighborhood contexts that reflect the full range of students’ daily activity spaces.

Second, the spatial clustering of unhealthful food outlets indicates that targeted, place-based strategies may be particularly effective. Zoning policies that regulate the density of fast food outlets near schools, incentives for healthy food retailers, and integration of food environment considerations into land-use planning may help reshape local food landscapes. Similar approaches have been recommended in prior research on built environment and food environment interventions [51].

Third, the identification of distinct urban and rural patterns highlights the need for context-specific approaches. In urban areas, efforts should focus on reducing exposure to unhealthful outlets and improving the relative availability of healthier options. In rural areas, strategies may prioritize increasing overall food access through infrastructure development, transportation solutions, and alternative food distribution systems such as mobile markets.

Finally, the findings emphasize the limitations of school-based nutrition interventions when implemented in isolation. External food environments may counteract improvements made within schools, underscoring the need for integrated approaches that combine environmental, policy, and educational strategies.

### 4.6. Strengths and Limitations

This study makes several methodological and substantive contributions. The multiscale spatial framework, integrating administrative units with network-based buffers, enhances the robustness of findings and addresses limitations associated with single-scale analyses. The integration of multiple complementary measures of food environments—including relative healthfulness, availability, accessibility, and spatial structure—provides a more comprehensive assessment than approaches relying on a single metric. In addition, the use of network-based walking-time buffers improves the behavioral relevance of accessibility measures.

The study also benefits from statewide coverage, allowing for the identification of broad spatial patterns while capturing localized variation through fine-scale analysis. The explicit focus on school-centered food environments further strengthens the relevance of findings for public health policy.

Several limitations should be considered. Although multiple geographic scales were examined, alternative definitions of neighborhoods and spatial contexts may yield different estimates of food environment exposure. The negative binomial regression analyses were descriptive in nature and did not adjust for potentially relevant contextual factors, including population density, socioeconomic characteristics, and land-use patterns. Consequently, the observed urbanicity differences should not be interpreted as independent causal effects of urbanicity itself. Furthermore, the regression analyses did not explicitly account for potential spatial dependence among schools. Future studies could employ spatial regression approaches and residual spatial autocorrelation diagnostics to further evaluate these relationships. GIS-based measures represent potential rather than actual exposure and do not capture individual behavior or activity patterns. The use of secondary commercial datasets may introduce inaccuracies due to omissions or misclassification of food outlets. The binary classification of outlets into “healthy” and “unhealthful” simplifies complex nutritional realities. Finally, other dimensions of food access, such as affordability and in-school food environments, were not examined.

### 4.7. Future Research Directions

Future research should extend this work by incorporating longitudinal designs to assess changes in school neighborhood food environments over time and their relationship to diet-related health outcomes. Integrating individual-level data on dietary behavior and mobility patterns would improve understanding of how environmental exposure translates into actual consumption. Additional research is also needed to evaluate the effectiveness of policy interventions aimed at modifying school neighborhood food environments. Expanding similar multiscale analyses to other states or regions would further enhance the generalizability of findings.

## 5. Conclusions

This study provides a comprehensive, multiscale assessment of the retail food environment around public schools in Michigan by integrating measures of relative healthfulness, availability, accessibility, and spatial structure. The findings demonstrate that school neighborhood food environments are highly uneven, spatially structured, and predominantly obesogenic. Across all measures, clear and consistent urbanicity gradients emerged—urban areas were characterized by greater availability, closer proximity, and stronger clustering of unhealthful food outlets, whereas rural areas exhibited lower availability and greater spatial isolation. Together, these patterns reveal a dual burden of food environment inequity operating through distinct mechanisms across the urban–rural continuum.

This study contributes to the literature by advancing a multiscale and multidimensional analytical framework that captures both regional disparities and localized variation in food environments. By integrating multiple spatial scales and exposure measures within a school-centered context, the findings demonstrate that no single metric or geographic scale is sufficient to characterize the complexity of food environments. The convergence of high availability, accessibility, and clustering of unhealthful food outlets—particularly around urban schools—highlights the extent to which environmental conditions may shape students’ dietary behaviors and reinforce obesogenic exposures.

From a public health perspective, these findings underscore the need for coordinated, place-based strategies that extend beyond the immediate school environment. In urban areas, policy approaches may include zoning regulations to limit the concentration of fast food outlets near schools and incentives to increase access to healthier food options. In rural areas, strategies should focus on improving overall food supply. More broadly, the results highlight the limitations of school-based interventions when external food environments remain unchanged, emphasizing the importance of integrated, cross-sectoral approaches.

Although this study focuses on Michigan, the observed multiscale patterns and urban–rural disparities are likely relevant to other regions with similar spatial and socioeconomic characteristics. These spatial patterns have important implications for children’s dietary behaviors and long-term health outcomes. The findings reinforce the importance of incorporating multiscalar perspectives in food environment research and public health planning. Ultimately, improving school neighborhood food environments will require addressing both the structural and spatial dimensions of food access to support healthier dietary behaviors and reduce long-term health risks among children.

## Figures and Tables

**Table 1:** Urban–rural differences in outlet availability (counts).

Outlet Type	Buffer	IRR (Urban vs. Rural)	95% CI	*p*
Fast food restaurants	10 min	2.27	2.05–2.51	<0.001
	15 min	2.45	2.25–2.66	<0.001
	20 min	2.80	2.59–3.03	<0.001
Convenience stores	10 min	1.25	1.08–1.43	0.002
	15 min	1.56	1.39–1.74	<0.001
	20 min	1.87	1.69–2.06	<0.001

IRR = incidence rate ratio; values > 1 indicate higher outlet availability in urban vs. rural schools. Urbanicity is defined using the U.S. Department of Education locale classification. Models were estimated using negative binomial regression. 95% CIs > 1 indicate statistical significance. *p*-values are from likelihood ratio tests.

**Table 2:** Differences in outlet availability (counts) across urbanicity categories.

Outlet Type	Buffer	City IRR	Suburban IRR	Town IRR	*p*	Significant Comparisons
Fast food restaurants	10 min	4.83	2.77	2.93	<0.001	City > Town, Suburban > Rural
	15 min	5.47	3.41	3.44	<0.001	City > Town, Suburban > Rural
	20 min	7.05	4.33	4.13	<0.001	City > Suburban, Town > Rural
Convenience stores	10 min	1.79	1.07	1.25	<0.001	City > Town, Suburban > Rural
	15 min	2.31	1.39	1.41	<0.001	City > Town, Suburban > Rural
	20 min	3.04	1.74	1.67	<0.001	City > Suburban, Town > Rural

IRR = incidence rate ratio; rural is the reference category. Values > 1 indicate higher outlet availability relative to rural schools. Urbanicity categories follow the U.S. Department of Education locale classification. Negative binomial models were used. Pairwise comparisons are Bonferroni-adjusted; only significant results (*p* < 0.05) are shown.

**Table 3:** Urban–rural differences in walking time (minutes) to the nearest unhealthful food outlet.

Outlet Type	Urban (Mean ± SD, *n* = 2095)	Rural (Mean ± SD, *n* = 1435)	*t* (*df*)	*p*	Cohen’s *d*
Fast food restaurants	15.0 ± 10.3	38.5 ± 52.7	−16.64 (*df* = 1509.3)	<0.001	−0.68
Convenience stores	21.2 ± 15.1	48.9 ± 63.2	−16.29 (*df* = 1546.3)	<0.001	−0.66

Values are mean walking time (minutes ± SD); lower values indicate greater accessibility. Urbanicity (urban vs. rural) is defined using the U.S. Department of Education locale classification. Welch’s *t*-test was used. Cohen’s *d* represents the standardized mean difference.

**Table 4:** Differences in walking time (minutes) to the nearest unhealthful food outlet across urbanicity categories.

Outlet Type	City (Mean ± SD, *n* = 792)	Suburban (Mean ± SD, *n* = 1303)	Town (Mean ± SD, *n* = 419)	Rural (Mean ± SD, *n* = 1016)	Welch’s *F* (*df*_1_, *df*_2_)	*p*	*ηp* ^2^	Significant Pairwise Differences (Games–Howell)
Fast food restaurants	11.8 ± 7.1	16.9 ± 11.4	15.3 ± 10.9	48.1 ± 59.7	164.11 (3, 1455.2)	<0.001	0.171	City < Town < Suburban < Rural
Convenience stores	16.4 ± 9.7	24.2 ± 16.9	30.9 ± 37.7	56.4 ± 69.7	166.24 (3, 1330.4)	<0.001	0.128	City < Suburban < Town < Rural

Values are mean walking time (minutes ± SD); lower values indicate greater accessibility. Urbanicity categories (city, suburban, town, rural) follow the U.S. Department of Education locale classification. Welch’s ANOVA with Games–Howell post hoc tests was used. Reported pairwise differences are significant at *p* < 0.05. *ηp*^2^ denotes effect size.

## Data Availability

The data used in this study are available from publicly accessible sources and commercial providers. Access to commercial data is subject to licensing restrictions. Derived data are available from the corresponding author upon reasonable request.

## Abbreviations

CDC	Centers for Disease Control and Prevention
CEPI	Michigan Center for Educational Performance and Information
CV	Coefficient of variation
EDGE	Education Demographic and Geographic Estimates
Esri	Environmental Systems Research Institute
mRFEI	The modified Retail Food Environment Index
NAICS	North American Industry Classification System
NCES	National Center for Education Statistics
NNA	Nearest Neighbor Analysis
NNI	Nearest Neighbor Index
USDA	U.S. Department of Agriculture

## Appendix A

**Table A1:** Average nearest neighbor summary statistics for unhealthful food outlets.

Outlet Type	NNI *	*z*-Score	*p*-Value	Observed Mean Distance (m)	Expected Mean Distance (m)
Unhealthful outlets combined (*N* = 9745)	0.284	−135.19	<0.001	557.9	1963.6
Fast food restaurants (*N* = 7680)	0.248	−126.04	<0.001	549.0	2211.9
Convenience stores (*N* = 2065)	0.575	−36.98	<0.001	2450.9	4265.6

* NNI: Nearest Neighbor Index.

**Table A2:** Schools (*N* = 3530) located within 10 and 20 min walk of unhealthful food outlets.

Outlet Type	Within 10 min		Within 20 min	
	# of Schools	%	# of Schools	%
Fast food restaurants	1201	34.0%	2287	64.8%
Convenience stores	766	21.7%	1819	51.5%

**Table A3:** Unhealthful food outlets located within 10, 15, and 20 min walk of schools.

Outlet Type	Within 10 min		Within 15 min		Within 20 min	
	# of Outlets	%	# of Outlets	%	# of Outlets	%
Fast food restaurants (*N* = 7680)	2466	32.1%	4228	55.1%	5472	71.3%
Convenience stores (*N* = 2065)	704	34.1%	1092	52.9%	1348	65.3%

**Table A4:** Games–Howell Pairwise Comparisons of Mean Walking Time to the Nearest Unhealthful Food Outlet Across Urbanicity Categories.

Comparison	Fast Food Restaurants		Convenience Stores	
	Mean Difference (Minutes)	Adjusted *p*	Mean Difference (Minutes)	Adjusted *p*
City vs. Suburban	−5.10	<0.001	−7.77	<0.001
City vs. Town	−3.48	<0.001	−14.45	<0.001
City vs. Rural	−36.21	<0.001	−39.98	<0.001
Suburban vs. Town	1.63	0.044	−6.69	0.003
Suburban vs. Rural	−31.10	<0.001	−32.21	<0.001
Town vs. Rural	−32.73	<0.001	−25.52	<0.001

Values represent mean differences in walking time (minutes) between urbanicity categories. Negative values indicate shorter walking times for the first category listed in each comparison. *p*-values are from Games–Howell post hoc tests following significant Welch ANOVA results. Urbanicity categories were defined using the U.S. Department of Education locale classification (City, Suburban, Town, Rural).

**Table A5:** Median (Q1–Q3) walking times (minutes) to the nearest unhealthful food outlet by urbanicity category.

Outlet Type	City (*n* = 792)	Suburban (*n* = 1303)	Town (*n* = 419)	Rural (*n* = 1016)
Fast food Restaurants	10.7 (6.8–15.3)	14.1 (9.2–21.8)	13.1 (8.1–19.5)	26.0 (11.6–59.7)
Convenience stores	14.3 (9.6–21.0)	19.4 (12.3–32.2)	18.0 (10.9–30.8)	32.8 (12.6–79.4)

Values are reported as median (Q1–Q3), where Q1 and Q3 represent the 25th and 75th percentiles, respectively. Lower values indicate greater accessibility to unhealthful food outlets. Urbanicity categories (city, suburban, town, and rural) follow the U.S. Department of Education locale classification system. Median and interquartile range statistics are provided as a robustness check for the mean-based analyses reported in Table 3 and Table 4. The median-based results demonstrate patterns consistent with the mean-based analyses.

**Table A6:** Robustness checks for urban–rural differences in walking times to the nearest unhealthful food outlet.

Outlet Type	Levene’s *F*	*p*	Mann–Whitney *U*	*Z*	*p*
Fast food Restaurants	826.84	<0.001	1,052,600	−15.15	<0.001
Convenience stores	811.58	<0.001	1,126,603	−12.66	<0.001

Significant Levene’s tests indicate unequal variances between urban and rural schools. Mann–Whitney *U* tests were conducted as nonparametric sensitivity analyses and yielded conclusions consistent with the Welch *t*-tests reported in Table 3.
